# Ex vivo biomechanical evaluation of tissue construct strength in an equine colopexy model

**DOI:** 10.1111/vsu.14117

**Published:** 2024-07-08

**Authors:** Hannah M. Gaitan, Margaret C. Mudge, Alan S. Litsky, Andreia G. Arruda, Alison K. Gardner

**Affiliations:** ^1^ Department of Veterinary Clinical Sciences College of Veterinary Medicine The Ohio State University Columbus Ohio USA; ^2^ Departments of Orthopedics and Engineering College of Medicine, The Ohio State University Columbus USA; ^3^ Department of Veterinary Preventive Medicine College of Veterinary Medicine The Ohio State University Columbus USA

## Abstract

**Objective:**

To compare strength of left paramedian colopexies using various techniques in equine ex vivo models.

**Study design:**

Experimental study.

**Sample population:**

Equine cadavers euthanized for nongastrointestinal pathology (36 specimens derived from 9 horses).

**Methods:**

Colopexies were performed after euthanasia. Suture pattern (horizontal mattress vs. cruciate) and incorporation of dorsal sheath of the rectus abdominis (partial‐thickness) versus incorporation of dorsal and ventral sheath of the rectus abdominis (full‐thickness) were evaluated. Single cycle load to failure, work to peak load, stiffness, and mode of failure of colopexy tissue constructs were assessed.

**Results:**

Mean load to failure of all constructs ranged from 102.26 to 166.38 N. Partial‐thickness bites demonstrated a mean load to failure and standard deviation (SD) of 111.91 (35.88) N and 102.26 (30.06) N (*p* < .05) which was significantly lower than the mean and SD of full‐thickness bites (166.3 [72.42] N and 163.21 [51.40 N]), respectively. All full‐thickness bites regardless of suture pattern and over half of partial‐thickness bites failed at the colonic wall. There was no significant difference in load to failure compared to mode of failure.

**Conclusion:**

A stronger colopexy was achieved with a full‐thickness bite regardless of the suture pattern. The most common mode of failure was the colon wall.

**Clinical significance:**

Incorporating ventral and dorsal fascia of the rectus abdominus provided a stronger colopexy structure, which may necessitate a second incision or subcutaneous palpation of the needle when performing a colopexy. The lateral band of the colon failed in most constructs (77%) regardless of technique, which could weaken the colonic wall and risk colonic rupture.

## 1 INTRODUCTION

Large colon volvulus and large colon displacement contribute to 33%–42% of all exploratory celiotomies and can recur even after successful surgical correction in horses.^1^ While any horse may suffer a large colon malposition and require surgical intervention, this condition is far more common in periparturient mares.^1^, ^2^, ^3^, ^4^ In broodmares, the risk of recurrence of large colon displacement or volvulus has been reported to be approximately 15% after surgical intervention. The risk of a broodmare having a third event increases to as high as 80% if recurrence requires a second surgical correction.^1^, ^2^, ^3^ Colopexy and resection of the large colon are two surgical procedures described to prevent recurrence of large colon malposition.^1^, ^2^, ^3^, ^5^ Large colon resections are often elected if the large colon is edematous or severely compromised, but complications associated with resection of the large colon include dehiscence of the anastomosis site, peritonitis, endotoxemia, and in the most extreme cases death of the animal.^6^ Studies have shown that short‐term survival was 58%–74% and long‐term survival was 50% for horses undergoing large colon resections.^7^, ^8^, ^9^, ^10^, ^11^, ^12^, ^13^ Colopexy is an option if the colon is deemed viable which results in a cleaner and faster surgery and avoids colon resection. Survival to discharge and long‐term survival (1 year) after colopexy has been reported as >90% and 78%, respectively, and avoids some of the complications associated with large colon resection.^1^, ^4^

Numerous surgical techniques for equine colopexy have been described,^1^, ^2^, ^5^, ^14^, ^15^, ^16^ with the most commonly reported technique describing suturing the lateral tenial band of the left ventral colon to the left body wall.^2^, ^3^ The ventral (external) fascia of the rectus abdominus muscle is described as the stronger “holding” layer of paramedian celiotomy closure.^17^, ^18^ Techniques to ensure that the ventral rectus fascia is included in the colopexy include creating the initial celiotomy incision paramedian to midline to incorporate the colon in closure, performing a secondary paramedian skin incision, or bluntly dissecting the subcutaneous tissue away from the ventral rectus fascia to the left of the original incision.^2^, ^3^ The use of a second skin incision ensures both the dorsal and ventral rectus fascia have been incorporated into the colopexy and avoids the creation of dead space due to dissection of subcutaneous tissue. Alternatively, a second incision increases surgical time and may increase the risk of infection development. Another colopexy technique involves incorporation of the lateral band of the left ventral colon into the ventral midline closure.^16^ However, this poses a danger to the animal if a second celiotomy becomes necessary at any future timepoint, as inadvertent entry into the colon would occur if the surgeon approached the abdomen on ventral midline without knowledge of this prior procedure. A recent case series also reported that dehiscence of the colopexy at this site was noted in three mares who had recurrence of large colon volvulus, necessitating re‐laparotomy.^5^

In previous publications, the authors described a technique to avoid a second abdominal incision by elevating the body wall during suture placement and visualizing the needle passing subcutaneously without penetrating the skin.^19^ Although this technique should engage the dorsal and ventral rectus in most cases, in horses with a thicker body wall or when a smaller needle is utilized the ventral rectus may not be fully engaged. This led to our investigation of colopexy construct strength with thickness of bite as the main variable and suture pattern as a secondary variable.

The contribution of tissue layers and suturing techniques to the overall tissue strength of the colopexy construct has not been previously examined to our knowledge. Suture breakage or dehiscence of the colopexy could lead to recurrence of large colon displacement or volvulus. Initial strength of the colopexy construct is critical until the development of fibrous adhesions, as recurrence of displacement or volvulus can occur within 48 h of the initial event.^2^, ^5^, ^20^ More catastrophically, a reported complication of the technique is rupture of the large colon adjacent to the colopexy site, resulting in endotoxemia and death of the animal.^1^, ^2^ This complication usually occurs before a robust fibrous adhesion can form, with studies showing loss of 1.25%–6.8% of horses within 4–12 weeks of the initial colopexy procedure due to colonic rupture.^1^, ^4^

Evaluating load to failure as well as mode of failure of the equine colopexy site using different suture patterns (horizontal mattress vs. cruciate) and depth of suture bites (full‐thickness vs. partial‐thickness) may help elucidate the best surgical technique. Potential alterations to surgical technique may decrease the overall surgical time, reduce failure of the colopexy at the site of the colon, negate the need for a second paramedian skin incision, and provide a stronger colopexy. The objectives of this study were to compare a single cycle load to failure, stiffness, peak load, and mode of failure of multiple tissue constructs performed using two different suture patterns and two tissue thicknesses in an equine colopexy experimental model.

Interrupted cruciate and horizontal patterns as well as a simple continuous suture pattern have each been described in the equine literature. An interrupted cruciate and horizontal pattern were chosen for this study to evaluate if the suture orientation relative to muscle fibers of the rectus abdominus influenced strength of the colopexy. We hypothesized that the single cycle load to failure would not differ significantly between full‐thickness bites versus partial‐thickness bites. Our second hypothesis was that the mode of failure via body wall or colon wall would not differ between the different colopexy constructs.

## 2 MATERIALS AND METHODS

### 2.1 Sample population

Nine horses between 2 and 25 years of age and weighing between 450 and 550 kg were used in this study immediately after euthanasia. Horses were donated to The Ohio State University for the purpose of euthanasia for reasons other than gastrointestinal disease or cachexia, such as untreatable musculoskeletal or neurologic disease. Procedures performed on horses were approved by the institutional animal care and use committee (IACUC AUP# 2009A0015‐R5).

Sample size calculations were conducted using EpiTools.^21^ As preliminary data in horses were unavailable to investigators, estimates from Levine et al. were used.^22^ To detect a difference in mean tension of 1.58 kg (3.88 ± 0.82 kg within the partial‐thickness group and 5.46 ± 1.21 kg within the full‐thickness experimental group) using a 95% confidence interval and 80% power, a minimum of seven samples per experimental group was needed.

### 2.2 Specimen collection

**FIGURE 1:**
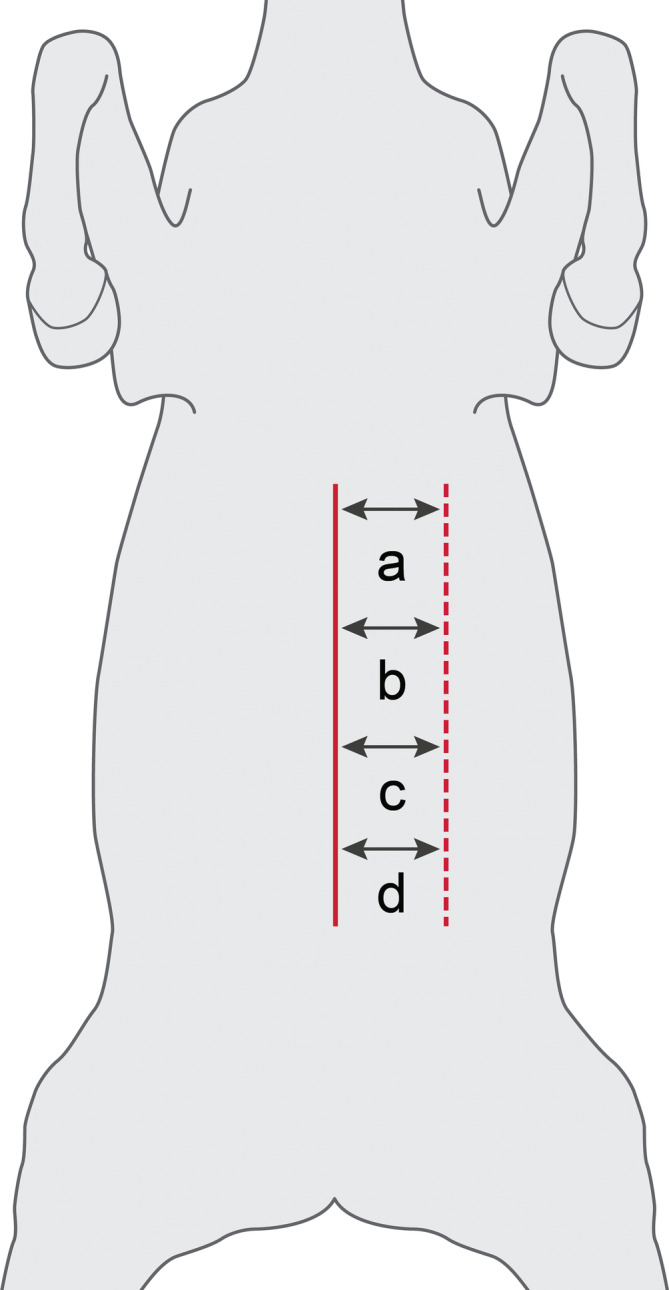
Illustrative example of mapping colopexy placement on the ventrum of a horse post‐euthanasia. Standardized placement of colopexy sutures was ensured by measuring and marking each cadaver after clipping the abdominal ventrum. A colopexy was performed through a ventral median incision (solid red line), with four different constructs performed over a length of 35 cm. Each lowercase letter designated a different variable (pattern/depth); order of variables in a cranial to caudal fashion were established and randomized using a Latin square. An incision only through the skin was made along the length of proposed colopexy to confirm full‐thickness bites (dashed red line). Each colopexy construct was performed exactly 6 cm from the linea alba (double arrowheads).

**FIGURE 2:**
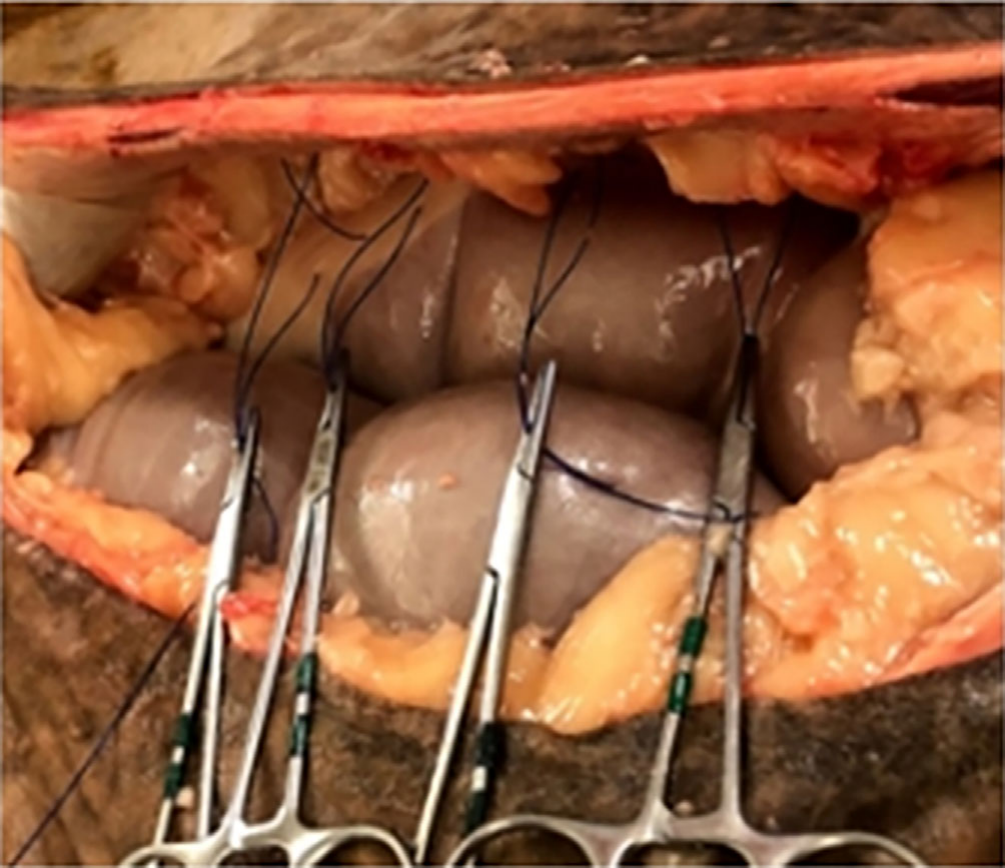
Colopexy suture placement prior to tying. This figure displays the four different colopexy structures (grasped with the four hemostatic clamps) preplaced by the same board‐certified surgeon prior to tying.

**FIGURE 3:**
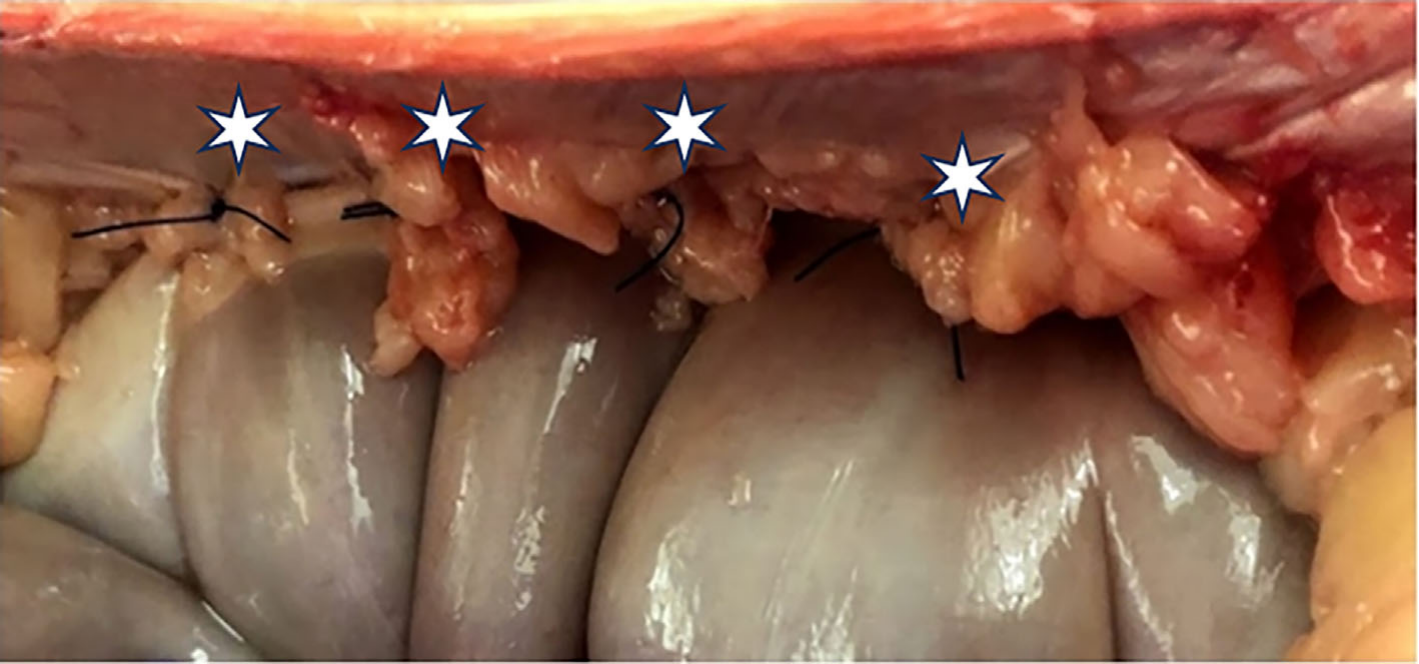
Colopexy in situ. The lateral band of the ventral colon was brought to the body wall after the surgeon tied and secured the four different colopexy constructs (white asterisk).

Four single suture colopexy constructs were performed on each horse in the same fashion and were assigned order of construct type (suture pattern and thickness) in a cranial to caudal direction for a total of 36 colopexy constructs consisting of colon, suture, and body wall in a randomized Latin square design. Immediately following euthanasia using an overdose of pentobarbital sodium and phenytoin sodium (Euthaphen, Dechra Veterinary Products, Overland Park, Kansas), horses were placed into dorsal recumbency on a standard operating table to replicate a standard exploratory laparotomy with colopexy. For each horse, the ventral abdomen was clipped, measured, and marked as shown in Figure 1. The abdomen was opened using a standard ventral midline celiotomy technique. A left paramedian skin incision was then made to ensure proper bite thickness (full‐thickness engaging the dorsal and ventral rectus fascia or partial‐thickness engaging only the dorsal rectus fascia) was achieved for the four different colopexy constructs. Four different colopexy constructs (Figure 2) were performed on each horse by the same ACVS board‐certified surgeon with 18 years of experience. The four colopexy constructs were as follows: (1) cruciate suture with partial‐thickness bites, (2) cruciate suture with full‐thickness bites, (3) horizontal mattress with partial‐thickness bites, and (4) horizontal mattress with full‐thickness bites. Size 1 polypropylene on a 65 mm ½ circle taper needle (Prolene, Ethicon Inc., Raritan, New Jersey) was used for all colopexy constructs. The distance between suture bites in the colopexy and distance the colopexy was from midline were standardized for each horse with each colopexy site made 6 cm to the left of the ventral midline. Partial‐thickness bites were performed engaging the submucosal layer of the left lateral band of the colon for each colopexy construct. The four different colopexy constructs were performed over 30 cm with approximately 7 cm between each construct to allow for adequate tissue dissection for individualized biomechanical testing. Each suture pattern (cruciate and horizontal mattress) was 3 cm × 3 cm. Craniocaudal order of the four constructs along the abdominal wall was randomized using Latin square between horses to account for changes in the thickness of the linea alba.^23^ Suture bites for all four constructs were measured and preplaced using the above requirements before tying (Figure 2). An assistant elevated the left body wall and the surgeon tied knots adjacent to the colon and dorsal rectus at each of the four constructs to bring the lateral band of the colon firmly against the body wall as displayed in Figure 3. Each colopexy construct was then dissected from the cadaver for immediate biomechanical testing. Colopexies were performed and dissected from the cadaver within 1 h of euthanasia. Constructs were transported on ice in a Styrofoam cooler to the biomechanical testing facility within 10 min and tested by the same individual within 30 min of collection.

### 2.3 Biomechanical testing

**FIGURE 4:**
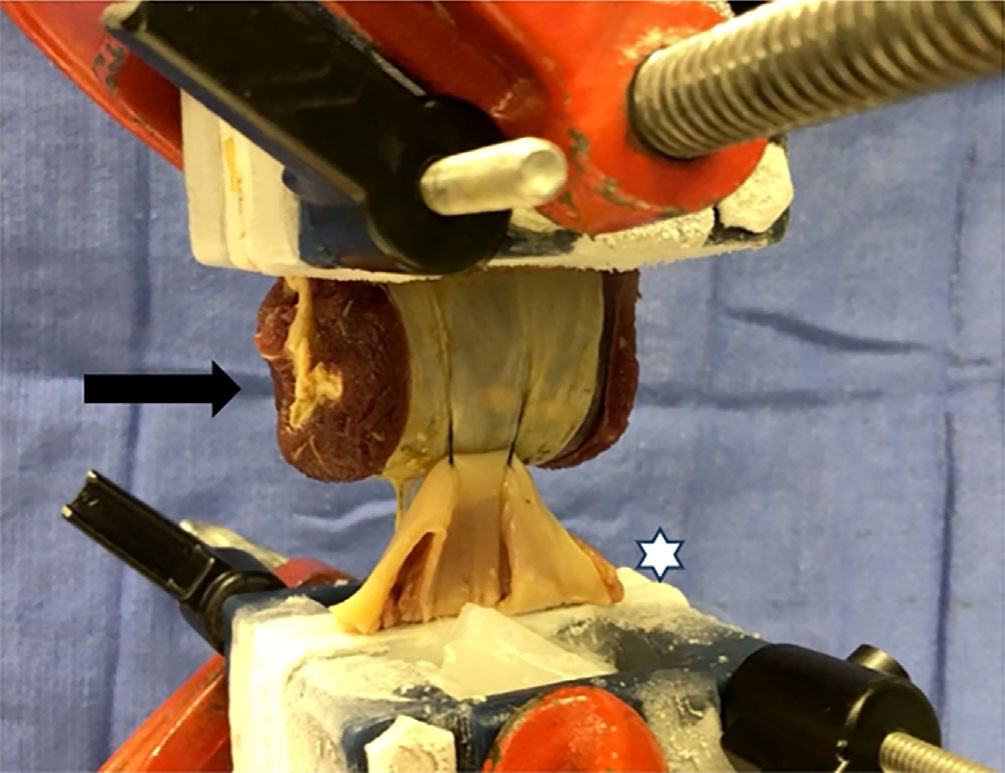
Biomechanical testing of each construct. The body wall was placed in one clamp of the system (black arrow) and the colon in the other (white asterisk). Cryo‐grips, cooled by dry ice, were used to secure the tissues and to prevent slippage in the grips. Displacement took place in a vertical plane.

All tissue constructs were tested under the same conditions and methods. Biomechanical testing was performed on each construct using a servohydraulic materials test system (MTS Bionix 858, MTS Corp., Eden Prairie, Minnesota) (Figure 4). This was similar to a procedure previously described in a canine urethral resection and anastomosis model.^24^ The same individual performing all biomechanical testing was blinded to the type of construct being evaluated. Custom cryoclamps outfitted with dry ice compartments to locally freeze tissue were used to firmly grasp tissue in an area separate from the individual soft tissue construct being tested, with the body wall positioned in the proximal clamp and the colon wall positioned in the distal clamp (Figure 4). Care was taken to ensure the soft tissue was not damaged and held in a neutral position prior to biomechanical testing. Additionally, careful attention was made to ensure the same amount of tissue was grasped on either side of the clamp and the cryoclamps were positioned approximately 6–8 cm away from the colopexy construct. The suture line between the colon wall and the abdominal wall was loaded in tension with a constant rate of displacement of 0.5 mm/second in a vertical plane to obtain load to failure, stiffness, work to peak load, and mode of failure of each of the four tissue constructs. The first three variables were calculated using a load deformation curve. Load (Newtons), and axial displacement (mm) were recorded every 0.1 s (10 times/s) to determine load deformation curve. Load to failure (Newtons) or ultimate strength of the construct was calculated from load‐deformation curves produced for each separate construct and was defined as the highest load (peak load) along the curve before tissue or suture was disrupted. Work to peak load (N × mm) was obtained by triangulating the area under the entire load deformation curve to the point of the peak load for each construct. Stiffness (N/mm) of the curve, exhibited by the most linear portion of the load deformation curve, was obtained by dividing the stress by the strain in the elastic portion of the graph. The mode of failure for each construct was defined as the location at which the construct failed and was categorized as follows: failure of the suture, failure of the colon wall, or failure of the body wall. Mode of failure was visually assessed for each construct and recorded during each test. In addition to each soft tissue construct, size 1 polypropylene suture was tested alone to evaluate load to failure or strength of the suture by creating a single loop around one arm of the servohydraulic test system clamp tied with a 6‐throw knot on the opposing clamp. All biomechanical testing and calculations were performed blindly to the suture pattern and partial‐ or full‐thickness bites conducted.

### 2.4 Statistical analysis

Statistical analysis was conducted using STATA 17 (College Station, Texas). Frequency (%) was calculated for binary and categorical variables, and basic statistics including mean (SD), minimum and maximum were calculated for numerical variables. Four different multivariable mixed linear models were created for each of four outcomes; namely load to failure, work to peak load, stiffness, and mode of failure. All models included horse as a random effect to account for the repeated contributions of specimens within animals. Type of suture, thickness, and the interaction between those two variables were added as main predictors. Failure modes for the different constructs were descriptively analyzed. Statistical significance was declared at *p* < .05.

## 3 RESULTS

### 3.1 Biomechanical properties

#### 3.1.1 Load to failure

**FIGURE 5:**
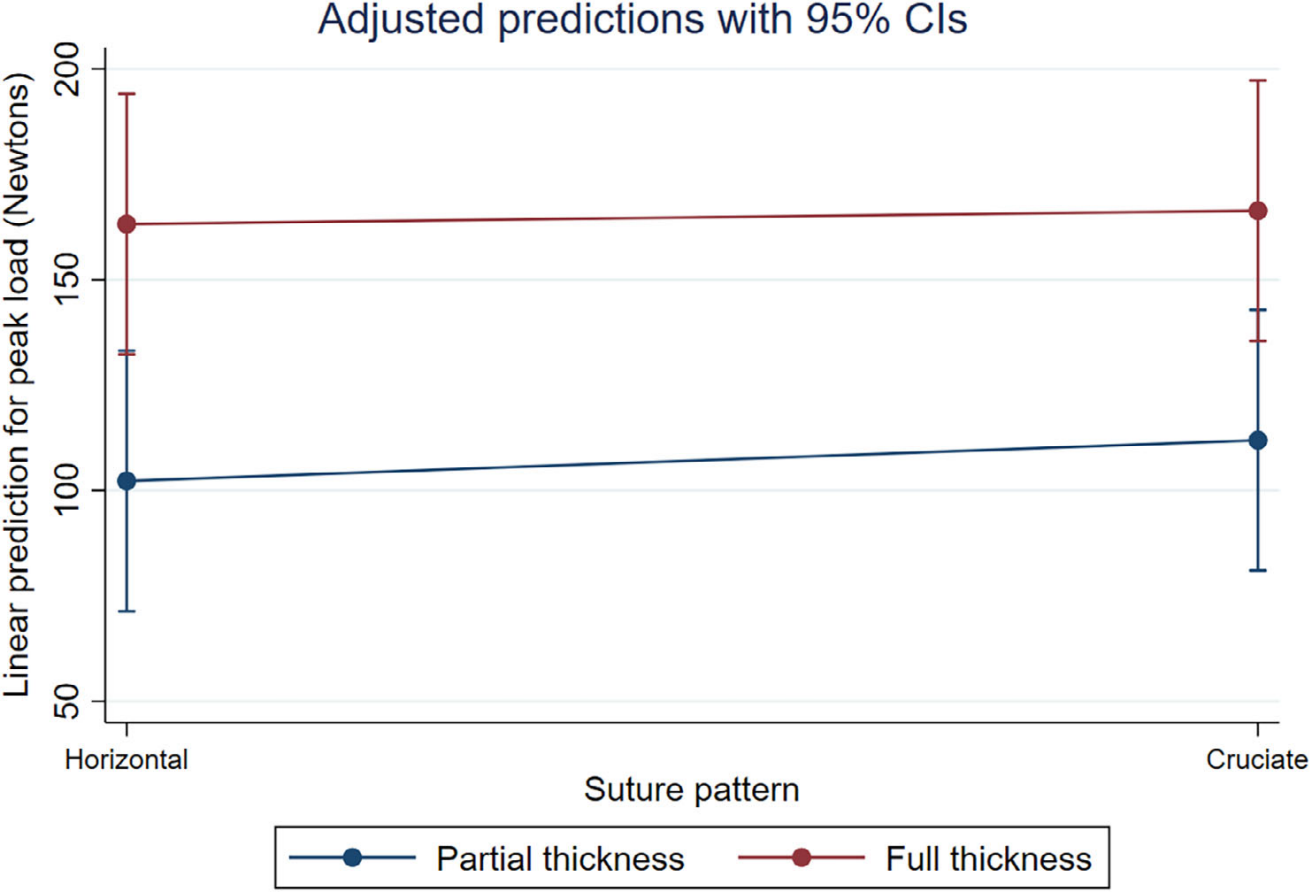
Results from multivariable model depicting a significant interaction between suture pattern and thickness for the outcome peak load. Full‐thickness bites had a statistically significant higher peak load failure (y‐axis) regardless of suture technique (x‐axis) when compared to partial‐thickness bites.

The reported mean (SD) load to failure values for the full‐thickness constructs were 166.38 (72.42) N and 163.21 (51.40) N for the cruciate and horizontal suture full‐thickness patterns, respectively. The constructs using partial‐thickness bites required numerically lower force to achieve failure with mean (SD) values reported at 111.91 (35.88) N and 102.26 (30.06) N for the cruciate, and horizontal partial‐thickness bites, respectively. The results indicated a statistically significant interaction between suture type and thickness on load to failure (*p* = .0005; Figure 5). Specifically, there were significant higher peak to load values for full‐thickness constructs compared to partial‐thickness constructs for both testing conducted within the horizontal (*p* = .0035) and cruciate (*p* = .0091) suture patterns. There was not a statistical difference in load to failure between cruciate and horizontal suture pattern in either full‐thickness or partial‐thickness bites.

#### 3.1.2 Stiffness

**FIGURE 6:**
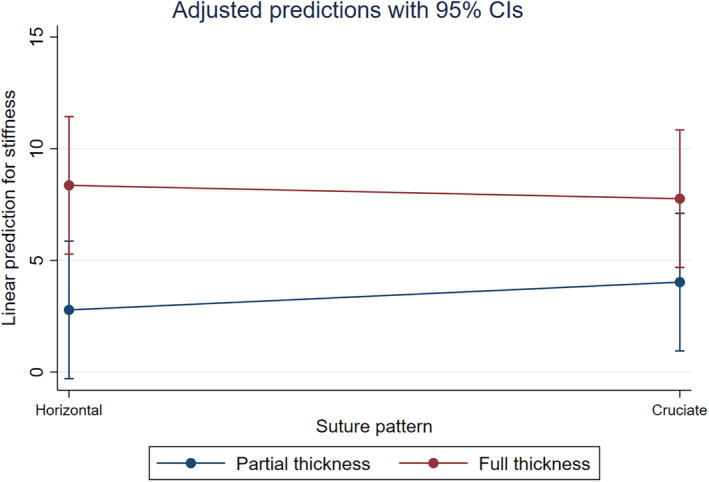
Results from multivariable model depicting a significant interaction between suture pattern and thickness for the outcome stiffness. Full‐thickness bites had a significantly higher stiffness (y‐axis) for the horizontal suture pattern (x‐axis) when compared to partial‐thickness bites.

There was a significant interaction between suture pattern and thickness when analyzing stiffness (Figure 6). The full‐thickness technique was strong with both suture patterns. Specifically, there was a significant difference within the horizontal suture pattern, with the full‐thickness construct being more resistant to deformation than the partial‐thickness construct with mean (SD) values reported at 7.76 (7.45) N/mm compared to 4.02 (2.86) N/mm. There was a statistical trend for the full‐thickness constructs to also be more resistant to deformation compared to the partial‐thickness within the cruciate suture pattern specimens with mean (SD) values reported at 2.79 (1.03) N/mm compared to 8.36 (5.93) N/mm.

#### 3.1.3 Work to peak load

The results indicated no significant differences in work to peak load when analyzing thickness, suture pattern and their interaction. The full‐thickness technique with mean (SD) values reported as 2215.33 (1105.70) N × mm and 2423.89 (1302.47) N × mm were insignificantly different (*p* = .953) when compared to the partial‐thickness technique 2581.33 (2792.54) N × mm and 1993.00 (1125.20) N × mm. Additionally, the cruciate suture pattern technique with mean (SD) values reported at 2215.33 (1105.70) N × mm and 2581.33 (2792.54) N × mm were insignificantly different (*p* = .729) when compared to the horizontal pattern technique 2423.89 (1302.47) N × mm and 1993.00 (1125.20) N × mm.

#### 3.1.4 Mode of failure

Constructs were reported to fail along either the colon wall, abdominal wall, or the suture. Approximately half 55.5% (10/18) of the partial‐thickness constructs failed at the colon, 27.7% (5/18) at the abdominal wall, and 16.7% (3/18) at the suture. In contrast, 100% (18/18) of full‐thickness constructs failed at the colon. Of the 27.7% (5/18) of partial‐thickness constructs that failed along the abdominal wall, all involved a cruciate pattern as no horizontal patterns failed along the abdominal wall. When examining constructs that failed at the suture 16.7% (3/18) of the partial‐thickness constructs failed at the suture; 5.5% (1/18) used a cruciate pattern and 11.1% (2/18) used a horizontal pattern.

## 4 DISCUSSION

In this study, we demonstrated that full‐ and partial‐thickness constructs regardless of the suture technique used were more likely to fail at the colonic wall instead of the abdominal wall. Additionally, full‐thickness bites were determined to be stronger than partial‐thickness bites when a single cycle load to failure was evaluated. Suture pattern (horizontal and cruciate mattress) was not a significant factor when evaluating the load to failure and mode of failure of colopexy constructs.

Several studies have been performed to evaluate the biomechanics of incisional gastropexy in canines,^25^, ^26^ but to our knowledge have not been conducted in colopexies in the horse. Load to failure, work to peak load, and stiffness are the most critical biomechanical properties of soft tissue and bony structures and can be assessed by external forces (loads) on the individual constructs.^27^ The load deformation curve is used to determine deformation of the tissue construct when a constant load is applied in a known direction. An additional metric, that is the distance of distraction at the point of failure multiplied by the force applied to cause failure, is termed work to peak load. The mechanical properties of the given construct can be determined based on the data represented by the load deformation curve. The point of failure on the curve represents the highest external force (peak load) at which the construct will fail, thereby determining the maximal strength of the construct. The elastic region of the load deformation curve is indicated by the upward slope of the curve and is defined as the region at which the structure will return to its original shape after unloading despite the external forces applied. Furthermore, the slope of the elastic region is defined as the construct's stiffness. Mode of failure is determined by visual assessment of the location of failure on the construct compared with the time at which the construct reaches its point of failure, which is important when two different tissues joined by suture are being assessed, as in the case of a colopexy. A thorough understanding of these mechanical properties applied to a given construct can aid in surgical decision making to produce the strongest construct possible.

Repeatable biomechanical testing of soft tissue structures, especially those made of multiple tissue types and layers, is limited by the ability of the biomechanical clamps to reliably hold tissue without affecting measurements. Previous reports of biomechanical testing of equine body wall focused mostly on strength of abdominal wall closure.^28^, ^29^, ^30^ Use of cryoclamps have been reported in equine body wall testing, with the tissue of interest remaining soft, pliable, and remote from the frozen, clamped section.^29^ Our group used dry ice in customized cryoclamps as opposed to running liquid nitrogen over the gripped tissue within the clamps, which obviated risk of inadvertent liquid nitrogen contact with the construct. This study showed feasibility of cyclic biomechanical testing of single suture equine colopexy constructs using cryoclamps.

Colonic location of the suture was chosen based on a previous report indicating that suturing 35 cm of the lateral free band of the left ventral colon to the abdominal wall (approximately 6 cm from midline) prevented displacement of the large colon at both the sternal and diaphragmatic flexures as well as the base. Another technique reported in this study described suturing the medial free band of the left and right colon together followed by suturing 8 cm of the lateral band of the left colon to the body wall. This technique was rejected due to the fact that some horses in this group lost enough condition to warrant euthanasia, and it did not prevent manual creation of a volvulus at the colon base.^31^ The colopexy sites performed here are the most commonly described for a paramedian colopexy in current literature and in this experimental study.^1^, ^31^

All of the full‐thickness and the majority of partial‐thickness colopexies failed at the colonic wall during biomechanical testing regardless of the suture pattern used or the depth of needle bite, with some partial‐thickness colopexies failing at the body wall and an even smaller percentage failing due to suture breakage. Interestingly, previous reports of testing ex vivo suture strength in body wall reported that suture was more likely to break before failure of the body wall occured.^28^, ^30^ However, body wall failure was reported as the major cause of dehiscence in a recent retrospective study in horses having received exploratory celiotomy.^32^ Failure along the colon could lead to fatal complications if the colon wall is weakened, increasing risk of rupture, whereas failure along the abdominal wall will result in loss of the colopexy and increase the risk for displacement. While a strong construct is needed to prevent colopexy failure, it may be beneficial to further investigate techniques that promote failure at the body wall without sacrificing strength.

Peak load of partial‐thickness constructs was significantly lower compared to full‐thickness constructs, while peak load at failure was not statistically different when comparing suture patterns between the two constructs. However, average load to failure in all constructs (102.26–166.38 N) regardless of construct type was stronger than reported mean breaking strength of single suture body wall constructs in a linea alba biomechanical testing model (71.4 N at the cranial abdomen and 101.4 N at the umbilicus)^28^ and in a study reporting on strength of body wall closure 2 weeks after body wall closure using cruciate sutures (87.7 N).^29^ Therefore, while full‐thickness patterns resulted in a stronger ex vivo interrupted pattern colopexy construct, the strength of a partial‐thickness colopexy may be sufficient to maintain strength of the colopexy under normal clinical conditions. The differences in load to failure between these two biomechanical testing studies is also interesting, as it infers that the load required to disrupt a colopexy may be greater than that to disrupt ventral midline closure. However, further testing would have to be performed to confirm this, as testing parameters were not equivalent between the two studies.

Stiffness may be another important variable to consider in optimal colopexy construction. An increase in stiffness is directly related to an increase in energy storage before failure, or the “breaking energy.”^29^ In our study, full‐thickness constructs had a higher stiffness than partial‐thickness. It is unknown if lower stiffness, or increased deformability relative to load applied, would result in a greater likelihood of construct failure in an in vivo construct. Recurrence of volvulus and displacement of the large colon in the immediate postoperative period would greatly stress the colopexy, and partial‐thickness suture bites may result in a higher risk of colopexy failure at the body wall or colon wall due to lower breaking energy. Cyclic loading of the colopexy may also stress a construct with lower stiffness. In vivo, postoperative feeding and conservative return to work and turnout schedules should be taken into consideration to lower cyclic stresses on the colopexy. Feeding regimens similar to post‐ large colon resection such as slow refeeding especially in regard to addition of nonstructural carbohydrates should be considered. Minimizing strain on the colopexy site through mitigating colonic distension will promote the formation of fibrous attachments. The body wall heals more slowly than permanent adhesion formation, so a return to work and turnout schedule for a horse receiving a colopexy will be the same as any horse having received a ventral midline celiotomy.

Interrupted horizontal mattress and cruciate patterns were compared in this study to evaluate whether orientation of suture relative to muscle fibers of the rectus abdominus influenced strength of the colopexy. No difference in strength was found between these two suture patterns, either regarding partial‐ or full‐thickness suture patterns. Further work to evaluate whether knot placement between the colon and body wall, as described in this study, has different biomechanical properties than a knot placed over the ventral fascia after performing a second paramedian skin incision is warranted.

The limitations of our study included a small population size but were comparable or larger based on samples in previous studies evaluating the strength of sutured soft tissue constructs in horses and other species.^25^, ^26^, ^28^, ^29^, ^30^, ^33^, ^34^, ^35^ Differences between colon and body wall thickness at different anatomical sites in the same animal was mitigated by randomizing construct type along the left ventrum from cranial to caudal, with each horse receiving each construct type. One large continuous colopexy site would be more realistic of a standard colopexy procedure and could be a focus of further research, as continuous suture lines better appropriate tension across the entire construct rather than interrupted sutures.

Size 1 polypropylene was chosen due to its previously described ability to more reliably remain intact long enough for adhesion formation from the body wall to the colon over some types of absorbable suture,^15^ although other suture materials have been reported.^1^ Future studies examining different suture types on the colon and abdominal wall are warranted. A 65 mm long ½ circle taper needle was used to perform the colopexies, as it would easily penetrate full‐thickness when needed even in animals with a healthy retroperitoneal fat layer while grasping significant colon and body wall tissue for incorporation into the construct. The taper needle allows the surgeon to feel the grasp of the submucosa (holding layer of viscera) in the colon without penetrating the lumen. Further investigation into type, length, and curvature of needle should also be performed to assess biomechanical properties associated with breadth of tissue grasped with each needle bite.

Finally, further studies are needed to determine the forces applied to a colopexy site in vivo. The movement and forces applied to the colon in a live horse are cyclical, such as forces from gas distention, continuous peristalsis, and forces from everyday movement and strenuous exercise (jumping and galloping). This study evaluated biomechanical properties of colopexy constructs under a single cycle load using continuous distraction, which may not mimic the stresses applied to a colopexy. This may mimic a force applied to the colopexy during a single event of colonic distension or movement but does not recreate cyclical loading and the shear forces applied to the construct during daily movement of the animal. With regard to healing, it is likely that a colopexy gains strength as formation of fibrous tissue occurs during healing, similarly to what has been shown in biomechanical testing of the equine linea alba, which has greater strength after 4 weeks of healing.^29^ Understanding the biomechanics of fibrous tissue would help us determine if the same results of this study hold true for matured colopexies.

## 5 CONCLUSION

Single cycle load to failure biomechanical testing is feasible in an equine abdominal wall and colon wall colopexy construct using custom‐made cryoclamps. The findings of this study suggest the clinical importance of incorporating both the ventral and dorsal rectus fascia in order to provide the strongest colopexy construct. While power calculations were performed to establish a sample size of >7 animals, the data used for these calculations were from a study on dog gastropexy^22^ using a single‐cycle load to failure model similar to that in this study. The significant difference between full‐thickness and partial‐thickness load to failure in this study on equine tissue provide data to be used for sample size calculation and protocol development in future colopexy studies. Although partial‐thickness colopexy constructs were observed to be as strong as ventral midline closure in previously reported ex vivo studies, further research with a larger population size examining colopexy techniques with variables as suture material and needle type, as well as developing a method to test a continuous suture pattern ex vivo, are warranted. Results of this study indicated that full‐thickness bites were stronger than partial‐thickness bites when load to failure was evaluated and suture pattern was not a significant factor in load to failure or mode to failure.

## AUTHOR CONTRIBUTIONS

Gaitan HM, DVM: Contributed to design conception, sample acquisition and testing, and manuscript writing and editing. Mudge MC, VMD, DACVS (Large Animal), DACVECC (Large Animal): Contributed to design conception, sample acquisition and testing including performing all colopexies, and manuscript writing and editing. Litsky AS, MD, ScD: Contributed to design conception, testing, and manuscript writing and editing. Arruda AG, DVM, MS, PhD: Performed statistical analysis of data and contributed to manuscript writing and editing. Gardner AK, DVM, MS, DACVS (Large Animal), DACVECC (Large Animal): Contributed to design conception, sample acquisition and testing, and manuscript writing and editing. All authors give their final approval for publication of the study.

## CONFLICT OF INTEREST STATEMENT

The authors declare no conflicts of interest related to this report.
